# Overcoming the Three-Dimensional Complexity of Vulvar Defects: A Stepwise, Multi-Flap Approach

**DOI:** 10.1055/a-2606-9515

**Published:** 2025-07-11

**Authors:** Chuan-Han Ang, Walter J.X. Tan, Bien-Keem Tan, Khong-Yik Chew

**Affiliations:** 1 Department of Plastic, Reconstructive, and Aesthetic Surgery, Singapore General Hospital, Singapore; 2 Faculty of Medicine, University of Queensland, Brisbane, Queensland, Australia

**Keywords:** extramammary Paget's disease, squamous cell carcinoma, vulvar reconstruction

## Abstract

Complex vulvar defects are challenging owing to their three-dimensional characteristics. We introduce a combined flap approach to maximize the use of locoregional tissues. Four patients had defects involving the vaginal wall, anal canal, and perineum, with a size range of 108 to 157 cm ^2^ . The outcomes were analyzed using a questionnaire regarding micturition, defecation, coital function, introitus opening, and aesthetics. For the vulva, the gluteal fold flap was the primary flap, which was augmented by the mons pubis rotation flap, gracilis muscle flap, pudendal thigh flap, and medial thigh VY advancement flap. The perianal skin and anal defects were covered by the gluteal fold and buttock VY advancement flaps. Patients' satisfaction scores were favorable on follow-up. Our multi-flap approach optimized the aesthetic and functional results of combined vulvar–anal defects.

## Introduction

Complex vulvar defects are challenging owing to their three-dimensional nature and proximity to the vaginal, urethral, and anal orifices. Our previous series outlined the subunit principle of vulvar reconstruction, where a maximum of two flaps were used. 1 This series highlighted that any reconstruction plan must provide external coverage and inner lining, and avoid disruption of critical structures. The purpose of this paper is to demonstrate the utility of three or more flaps to reconstruct more challenging defects. We define complex defects as those which are bilateral, involving both vaginal and anal orifices, and requiring three or more flaps to achieve a functional and aesthetically acceptable outcome.

## Cases

**Fig. 1:**
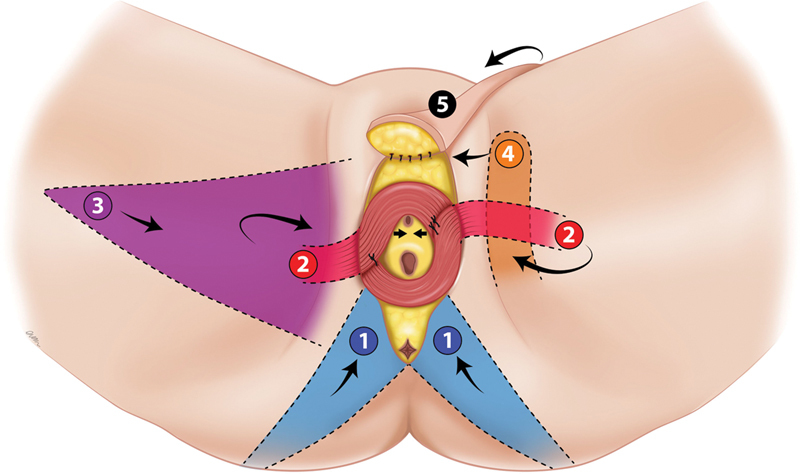
Types of local flaps used in complex vulvar defects. (1) Gluteal fold/buttock VY advancement flaps. (2) Gracilis muscle flaps. (3) Medial thigh VY advancement flap. (4) Pudendal thigh flap. (5) Superficial circumflex iliac perforator flap.

Four patients who underwent reconstruction for extensive vulvar defects from extramammary Paget's disease (EMPD; *n* = 3) and squamous cell carcinoma ( *n* = 1) resections were studied ( Table 1 ). The defects' sizes ranged from 108 to 157 cm ^2^ ; all involved the vaginal wall, anal canal, and perineum. We define the perineum as the patch of skin between the vulva and anus. The data collected were anonymized and recorded with informed consent from the patients. Patients' satisfaction after surgery was assessed using a questionnaire ( Appendix 1 [available in the online version only]), which was adapted from gynecological and urological sources. 2 3 4 The flaps included the gluteal fold VY advancement flap as the primary workhorse flap, 5 6 gracilis muscle flap, gracilis medial thigh VY advancement flap, mons pubis rotation flap, and buttock VY advancement flap ( Fig. 1 ). There were no major flap complications. The average follow-up period was 7 years. Two patients had superficial infections that responded to topical antifungals and intravenous antibiotics. The results of the patient satisfaction questionnaire are shown in Table 2 .

**Table 1:** Patient demographic data and outcomes

Case number	Age (years)	Diagnosis	Defect type	Defect size (cm ^2^ )	Type of reconstruction	Colostomy	Complications
1	64	EMPD	Bilateral, involving mons pubis, vulva, vagina, and anal verge	157	Bilateral gluteal fold VY advancement flaps, bilateral gracilis muscle flaps, and SCIP flap	Yes	Fungal infection (treated topically)
2	40	EMPD	Bilateral, involving vulva, introitus, and anal verge	108	Bilateral gluteal fold VY advancement flaps and pudendal thigh flap	Yes	Fungal infection (treated topically)
3	43	SCC	Bilateral, involving vulva, introitus, vagina, and anal canal	123	Bilateral gluteal fold VY advancement flaps and buttock VY advancement flap	Yes	Bacterial infection (treated with antibiotics)
4	60	EMPD	Bilateral, involving mons pubis, vulva, introitus, vagina, anal verge, and inner thigh	124	Bilateral gluteal fold VY advancement flaps, mons pubis rotation flap, and medial thigh VY advancement flap	No	Nil

Abbreviations: EMPD, extramammary Paget's disease; SCC, squamous cell carcinoma; SCIP, superficial circumflex iliac perforator.

**Table 2:** Patient satisfaction outcomes

Case number	Micturition	Defecation	Sexual function	Vaginal orifice	Scarring/Preservation of the vulvar subunits
1	Smooth, undeviated stream	Normal fecal caliber	Not applicable	Mildly constricted	Satisfied
2	Smooth, undeviated stream	Normal fecal caliber	Not applicable	Normal opening	Satisfied
3	Smooth, undeviated stream	Normal fecal caliber	Intercourse without lubrication	Normal opening	Satisfied
4	Smooth, undeviated stream	Normal fecal caliber	Not applicable	Normal opening	Satisfied

### Case 1

**Fig. 2:**
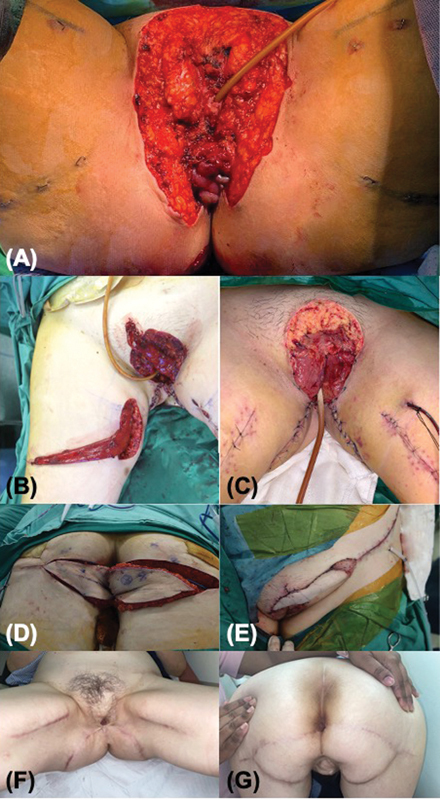
Patient of case 1. ( **A** ) Defect after EMPD resection. ( **B** ) The right gracilis muscle flap was mobilized. The left gracilis muscle was transposed medially to the defect. ( **C** ) The paired gracilis muscle flaps lining the introitus and vagina, before the application of skin grafts. The muscle flaps were sutured together at 6 o'clock to create the anovaginal septum. ( **D** ) Paired gluteal fold VY advancement flaps were mobilized to reconstruct the perianal skin and lower vulva. ( **E** ) A left SCIP flap was used to reconstruct the mons and the upper vulva. ( **F** ) Perineal view, 2 years postoperatively. ( **G** ) Buttock view, 2 years postoperatively. EMPD, extramammary Paget's disease; SCIP, superficial circumflex iliac perforator.

A 64-year-old patient presented with EMPD involving the mons pubis, vulva, vagina, and anal verge circumferentially ( Fig. 2 ). Five flaps were used in two stages to reconstruct the defect. Firstly, bilateral gracilis muscle flaps were mobilized to line the introitus and create a partition between the anus and the vagina. The muscle flaps were skin-grafted. Secondly, bilateral gluteal fold VY advancement flaps were used to reconstruct the perianal skin and the lower two-thirds of the vulva. The mons defect was temporarily covered with topical negative pressure wound therapy (NPWT) and prepped for closure at a second stage. Two weeks later, an extended super-thin superficial circumflex iliac perforator (SCIP) flap was raised to cover the mons pubis and the upper vulva bilaterally. Urinary and fecal diversion by means of a urinary catheter and loop colostomy kept the wounds clean. A month later, flap and scar revision were performed under local anesthesia. The colostomy was reversed after 3 months. Evaluation of her perineal function at 2 years showed smooth and undeviated urinary passage, normal fecal caliber, a smaller introitus, and no problems with feminine hygiene. The patient was satisfied with her physical and functional outcomes.

### Case 2

**Fig. 3:**
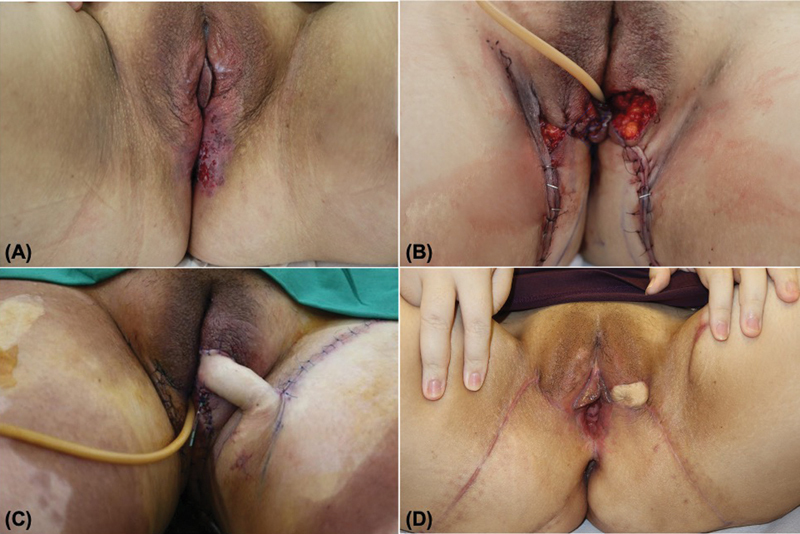
Patient of case 2. ( **A** ) EMPD involving the vulva and perianal skin bilaterally. ( **B** ) Paired gluteal fold VY advancement flaps were mobilized. ( **C** ) A pudendal thigh flap was used for the residual vulvar defect. ( **D** ) Result 6 months postoperatively. EMPD, extramammary Paget's disease.

A 40-year-old, obese patient presented with bilateral EMPD involving the vulva, introitus, and anal verge circumferentially ( Fig. 3 ). Following wide resection, three flaps were used in two stages to reconstruct the defects. First, the perianal skin and lower vulva were bilaterally reconstructed using paired VY advancement gluteal fold flaps. Then, a pedicled super-thin pudendal thigh flap was raised to cover the residual left vulva defect since the left gluteal fold flap could not reach the mid-vulva due to its adiposity. 7 She developed a postoperative cutaneous candidal infection, which responded to topical antifungals. As in case 1, she had a urinary catheter and loop colostomy to maintain hygiene. Evaluation at 6 months showed normal perineal function. She remained virgo intacta.

### Case 3

**Fig. 4:**
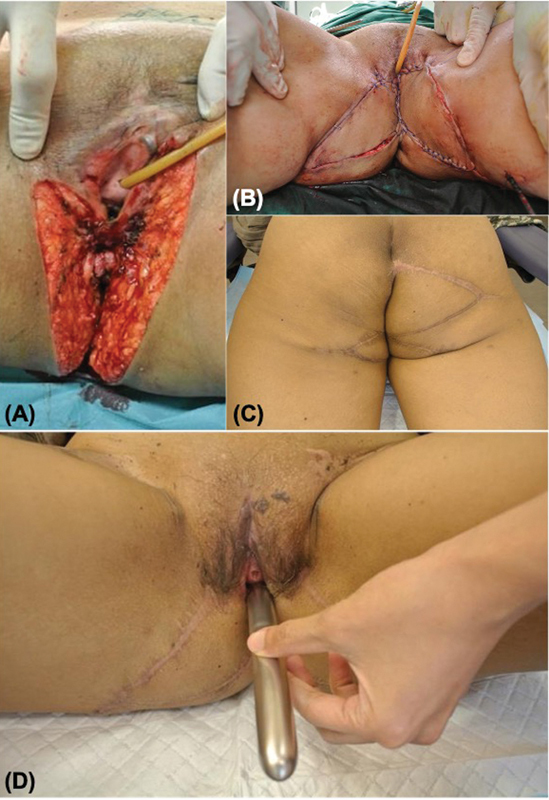
Patient of case 3. ( **A** ) Defect after resection of squamous cell carcinoma in situ. ( **B** ) Bilateral gluteal fold VY advancement flaps were mobilized to reconstruct the middle and lower vulva, and the vaginal wall. ( **C** ) A right buttock VY advancement flap was used to line the anal canal. ( **D** ) Result 7 years postoperatively. The dilator shows patency of the introitus and vagina.

A 43-year-old female presented with high-grade squamous intraepithelial carcinoma bilaterally, involving the vulva, introitus, vagina, and anal canal ( Fig. 4 ). She was sexually active. Furthermore, she had cervical and vaginal intraepithelial neoplasia III due to human papillomavirus 16. Tumor resection constituted laser ablation of cervical and vaginal lesions, a lower third vulvectomy that included the posterior vaginal wall, and circumferential excision of perianal skin including anal mucosa below the dentate line. Reconstruction was achieved in two stages. Firstly, the lower vulva and posterior vaginal wall were reconstructed using bilateral gluteal fold VY advancement flaps, which were crucial in recreating a partition between the vagina and anus. The anus was temporarily dressed with topical NPWT and the diverting colostomy kept it clean. Three weeks later, a right buttock VY advancement flap was used to resurface the anus. The folded “horns” of the advancement flap covered two-thirds of the anal canal, and the remaining third was left to heal by secondary intention. Her wounds were superficially infected with *Escherichia coli* and *Enterococcus faecalis* , which responded to systemic antibiotics. The colostomy was reversed 3 weeks after her anal canal had completely healed. Evaluation at 7 years showed normal perineal function. She had resumed sexual activity and was satisfied with her physical and functional outcomes.

### Case 4

**Fig. 5:**
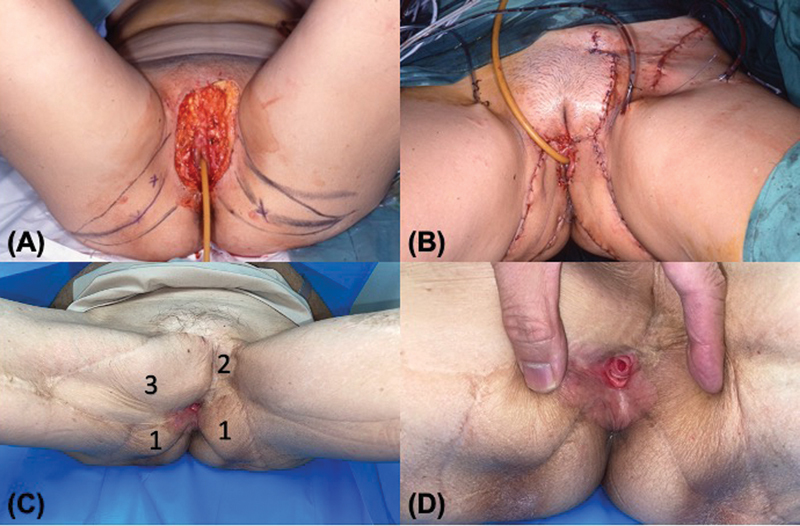
Patient of case 4. ( **A** ) Defect after resection of EMPD. Gluteal fold flaps were marked as shown. “X” denoted the loudest audible perforator signal detected by the Doppler probe. ( **B** ) Paired gluteal fold flaps and a mons pubis rotation flap completely cover the defects. ( **C** ) A gracilis medial thigh VY advancement flap was subsequently used for EMPD recurrence. (1) Gluteal fold flap; (2) mons rotation flap; and (3) gracilis medial thigh VY advancement flap. ( **D** ) Result 19 years postoperatively, showing a centrally located and patent urethral meatus. The vagina was patent and hidden. EMPD, extramammary Paget's disease.

A 60-year-old female presented with bilateral EMPD involving the mons pubis, vulva, introitus, vagina, anal verge, and the inner thigh ( Fig. 5 ). Following a wide resection, three flaps were used to reconstruct the defects. Initially, bilateral gluteal fold advancement flaps were used to reconstruct the lower vulva, and a mons skin rotation flap was mobilized to close the wound superiorly. Postoperatively, her wounds healed uneventfully, although her right margins were positive for EMPD. Six months later, she underwent a repeat resection over the right neo-vulva and coverage with a right gracilis medial thigh VY advancement flap. Evaluation 19 years postoperatively indicated normal perineal function. The patient was satisfied with her physical and functional outcomes.

## Discussion

The basis for our approach was to recreate native anatomical boundaries. When the defect extends from the vulva to the anus, the perineum must be reconstructed to avoid merging of the orifices. Otherwise, there would be no separation of excretory passages and vaginal secretions. Thus, simply approximating skin to close a defect was avoided.

From a lithotomy view, the vulva could be divided into three segments based on flap options 8 : the upper third, consisting of the mons pubis and the upper labia; the middle third, consisting of the labia proper; and the lower third, consisting of the lower vulva and perineum. The first segment was covered by the lower abdominal rotation flap or medial thigh VY advancement flap, while the last two segments were covered by gluteal fold flaps. 5 6 An adjacent area, constituting the perianal skin and anal canal, was covered using gluteal fold and buttock VY advancement flaps.

Case 1 was challenging because it necessitated the reconstruction of an anovaginal septum. The approximated gracilis muscle flaps in a “sphincter configuration” were useful for this purpose ( Fig. 2C ). The flaps were overlaid with skin grafts to line the vaginal wall. Paired gluteal fold VY flaps were then advanced over it to reconstruct the perianal skin, perineum, and lower vulva. A fifth flap from the left groin was raised to reconstruct the mons pubis and line the periurethral area. Attention was specifically directed towards preserving the periurethral lining to prevent scarring around the urethra. Although the patient was not sexually active, preservation of the introitus was necessary for maintaining vaginal hygiene. In case 2, apart from the paired gluteal fold flaps, an additional pudendal thigh flap was mobilized to preserve the bi-crescentic fullness of the labia majora. Sexual function took precedence for case 3, thus the paired gluteal fold flaps were prioritized for vulva and vagina wall reconstruction. After flap healing, the anus was reconstructed with a buttock advancement flap. Case 4 illustrated the utility of the medial thigh VY advancement flap in EMPD recurrence, since bilateral gluteal fold flaps were expended in the first operation.

Cho et al. 9 described the “three-directional” local flap approach for reconstruction of an extensive perineal defect using a pedicled gracilis myocutaneous flap for the deeper and bulkier portion of the defect, and other fasciocutaneous flaps for the thinner and shallower portions. Additionally, they highlighted advantages of using multiple flaps, such as being less prone to infection, and providing padding and pliability - qualities not seen with skin grafting. Single large flaps, including the anterolateral thigh flap 10 and deep inferior epigastric perforator flap, 11 have been used for such cases, but they are bulky and do little to mimic natural contours.

Topical NPWT was used liberally, as it promoted flap adherence to the wound bed. Achieving a seal was not difficult because of fecal and urinary diversion. This way, raw wounds were never left exposed as they can be easily colonized. Other nursing adjuncts included cage cradle nursing with an incorporated ventilation fan and in-bed off-loading exercises to prevent pressure injury.

In the rehabilitative period, the patients were taught perineal hygiene using a mirror, since sensory feedback is altered after surgery. Self-digital dilatation of the introitus and anus was initiated to promote tissue pliability. Reassurance was given that sexual activity could be resumed, aided by lubrication, after complete wound healing (case 3). From 3 to 6 months postoperatively, preparation for colostomy reversal entailed biofeedback exercises to improve coordination and relaxation of the anal sphincter. 12

### Conclusion

In this multi-flap approach, paired gluteal fold flaps were our workhorse flaps. Additional flaps were employed as necessary, with the aim to maximize functional and aesthetic outcomes.
