# Supplementary material for: Overcoming the Three-Dimensional Complexity of Vulvar Defects: A Stepwise, Multi-Flap Approach
Source: Arch Plast Surg. 2025 Jul 11;53(1):45–51. doi: 10.1055/a-2606-9515 (PMC12858315; doi:10.1055/a-2606-9515)
Supplement: Supplementary file 1 — Supplementary Material [file 10-1055-a-2606-9515-s24feb0031oa.pdf]

<u>Questionnaire Assessment</u>

This questionnaire focuses on potential complaints that you might have after your surgeries. We kindly ask you to answer the questions below in great detail.

1. Micturition
Do you currently experience any urinary complaints? Please tick the most appropriate option.

A. Do you have a smooth urinary stream? ▢
B. Do you have a narrowed urinary stream? ▢
C. Do you have a deviated or “interrupted” urinary stream? ▢
D. Do you experience urinary leakage, or have to strain to urinate? ▢

2. Defecation
Do you currently experience any bowel complaints? Please tick the most appropriate option.

A. Normal fecal caliber ▢
B. Narrowed fecal caliber ▢
C. Constipation (less than 3 bowel movements a week) ▢
D. Do you ever have to push on the vaginal wall or manually remove faeces to have a bowel movement? ▢

3. Subjective sexual function
Do you currently experience any sexual complaints? Please tick the most appropriate option.

A. Ability to have intercourse without lubrication ▢
B. Ability to have intercourse with lubrication ▢
C. Pain or bleeding during intercourse despite lubrication ▢
D. Unable to have intercourse ▢
E. Not applicable ▢

4. **Vaginal orifice (objective)**
How big is the hegar dilator that can be inserted into your vaginal orifice? Please tick the most appropriate option.

A. Normal coital function (hegar dilator size 15mm) ▢
B. Mildly constricted vaginal orifice (hegar dilator size 11mm) ▢
C. Moderately constricted vaginal orifice (hegar dilator size 7mm) ▢
D. Severely constricted vaginal orifice (hegar dilator size 3mm) ▢

5. Scarring/ preservation of vulvar subunits
Are you satisfied with the physical appearance of your perineum? Please tick the most appropriate option.

A. Patient satisfied with own physical appearance ▢
B. Minimal distortion of vulvar subunits ▢
C. Effacement of neighboring vulvar subunits ▢
D. Complete distortion and contraction of all vulvar subunits ▢
